# Density functional theory computation of the binding free energies between various mutations of SARS-CoV-2 RBD and human ACE2: molecular level roots of the contagiousness

**DOI:** 10.1016/j.heliyon.2022.e10128

**Published:** 2022-08-11

**Authors:** Serhan Yamacli, Mutlu Avci

**Affiliations:** a Nuh Naci Yazgan University, Department of Electrical-Electronics Engineering, 38090, Kayseri, Turkey; b Cukurova University, Department of Biomedical Engineering, 01330, Adana, Turkey

**Keywords:** SARS-CoV-2, Human ACE2, Density functional theory, Binding free energy

## Abstract

The receptor-binding domain (RBD) of SARS-CoV-2 attaches to the human ACE2 to initiate binding of SARS-CoV-2 to human cell and leads to the infection process afterwards. In this study, various mutations of SARS-CoV-2 spike RBD and human ACE2 complexes are investigated via density functional theory (DFT) computations to obtain binding free energies. The DFT computations are performed without fragmenting the interfaces to involve longer-range quantum mechanical interactions for improving accuracy. The vibrational free energies, van der Waals dispersion forces and basis set superposition error corrections are also included in the calculations. The results show that the absolute value of the binding energy of B.1.1.7 mutated spike RBD–ACE2 complex is more than five times higher than that of the original strain. The results of this study are expected to be useful for a deeper understanding of the relation of the binding free energies and the level of contagiousness.

## 1 Introduction

The severe acute respiratory syndrome coronavirus 2 (SARS-CoV-2) has been an emerging pathogen, which is the cause of the current coronavirus-19 disease (Covid-19) pandemic [1,2]. It is known that coronavirus family, including the severe acute respiratory syndrome coronavirus 1 (SARS-CoV-1), initiate the infection mechanism by binding to the host cell receptors [3]. Specifically, the receptor binding domains (RBDs) of the spike proteins of coronaviruses attach to human angiotensin-converting enzyme 2 (ACE2). The atomic-level understanding of the binding mechanisms of spike RBDs to human ACE2 are obviously of great importance for the design and implementation of vaccines and drugs [4,5].

Cryogenic electron microscopy (cryo-EM) and X-ray crystallography (XRC) play an important role for obtaining the molecular structure of biomolecules. These techniques have been utilized for the determination of the atomic structures of both spike proteins of coronaviruses [6] and spike protein—human ACE2 complexes [7]. Although cryo-EM and XRC provide important atomic coordinates within their resolution limits, these data do not include physical properties such as binding energies and charge densities. The data obtained from these molecular imaging methods therefore have to be processed to obtain functional characteristics and parameters. The molecular imagery of both SARS-CoV-1 and SARS-CoV-2 spike protein–human ACE2 complexes have been reported in the literature [8,9]. It is shown that various conformations of the spike proteins of these structures exist however, the prefusion conformation of spike proteins are the favourable form for the attachment to the ACE2 molecules of host cells [6]. Despite the structural similarities of SARS-CoV-1 and SARS-CoV-2, clear differences exist in their spike proteins. Moreover, various variants of the SARS-CoV-2 have also emerged during the Covid-19 pandemic and these variants are characterized by the mutations in their spike proteins [10, 11, 12, 13]. Therefore, it is important to obtain the molecular-level binding properties of the spike protein–ACE2 complexes to understand the binding mechanisms and binding energies. In the literature, the binding properties of SARS-CoV-1 spike–ACE2 and SARS-CoV-2–ACE2 complexes have been investigated using surface plasmon resonance (SPR) methods [9,14]. In addition, computational methods have also been used for the determination of the binding properties of these molecules such as molecular dynamics (MD) [15] and ab initio methods [16,17]. For example, a recent study reported the binding energies of SARS-CoV-1–human ACE2 as −10.81 kcal/mol and SARS-CoV-2–human ACE2 as −12.86 kcal/mol via classical MD simulations [15]. They have also computed the binding energies of the alpha and beta mutated SARS-CoV-2–human ACE2 complexes with MD computations as −14.66 kcal/mol and −13.52 kcal/mol, respectively [15]. Another paper presented the interaction of the spike protein of SARS-CoV-1 and human ACE2 molecule using accurate density functional theory (DFT) simulations by using fragmentation of the spike protein and ACE2 molecules to make the simulations tractable and used a fragmenting where a neighbourhood of 4.5 Å is used [16]. They have reported binding energies between −340.46 kcal/mol and −404.26 kcal/mol, depending on the basis set used. The same research group more recently studied the binding mechanism between SARS-CoV-2 and human ACE2 using the same fragmentation technique with a neighbourhood limit of 4.5 Å and reported a binding energy in the range of −45.02 kcal/mol and –98.60 kcal/mol including dispersion corrections [17]. In another study, molecular mechanics–generalized Born surface area (MM/GBSA) method was used to investigate the binding free energy of the wild and B.1.617 variant to the human ACE2 [18]. The MM/GBSA method was also used in [19] for the computation of binding free energies of the B.1.618 variant and human ACE2 and it is shown that B.1.168 variant sligtly alters the binding affinity. Similarly, the binding affinity of the B.1.1.529 variant is calculated using MM/GBSA method and it is shown that increasing mutations enhance the binding energy [20]. In another study, computational saturation mutagenesis is utilized to analyze 18354 spike protein mutations and 11324 ACE2 mutations where it is shown that D614G helps the spike protein to stabilize in 5703 strains [21]. The genomic variations in the structural proteins of SARS-CoV-2 have been investigated systematically in [22] and it is concluded that a slight change in sequence may lead to great changes in the pathogenesis of SARS-CoV-2.

In this work, we have studied the binding energies and binding mechanisms of the human ACE2 molecule to the spike proteins of the original SARS-CoV-2 strain, SARS-CoV-2 with N501Y mutant spike protein, SARS-CoV-2 with spike RBD having G485R mutation, SARS-CoV-2 P.1 variant spike glycoprotein and SARS-CoV-2 S-UK variant (B.1.1.7) with A570D and N501Y mutations, using DFT simulations. Our DFT calculations considered the RBD of spike proteins and human ACE2 molecule within 15 Å neighbourhood of the interface without fragmentation to include the effects of longer-range interactions in the binding energy. Moreover, we have employed counterpoise correction for basis set superposition errors (BSSE) during the energy calculations of spike protein–ACE2 complexes. In addition, van der Waals dispersion corrections together with the vibrational free energy calculations are also included for increasing the accuracy of the obtained binding energy values. Our results show that the absolute value of the binding energy of the SARS-CoV-2 S-UK variant (B.1.1.7) with A570D and N501Y mutations to the human ACE2 is more than five times greater than the absolute value of the binding energy of the original SARS-CoV-2 spike protein to human ACE2. We have also included the 2D and 3D partial charge variations before and after the attachment process, which are supposed to give a more complete picture of the binding mechanisms for vaccine or drug designing researchers.

## 2 Material and methods

**Figure 1:**
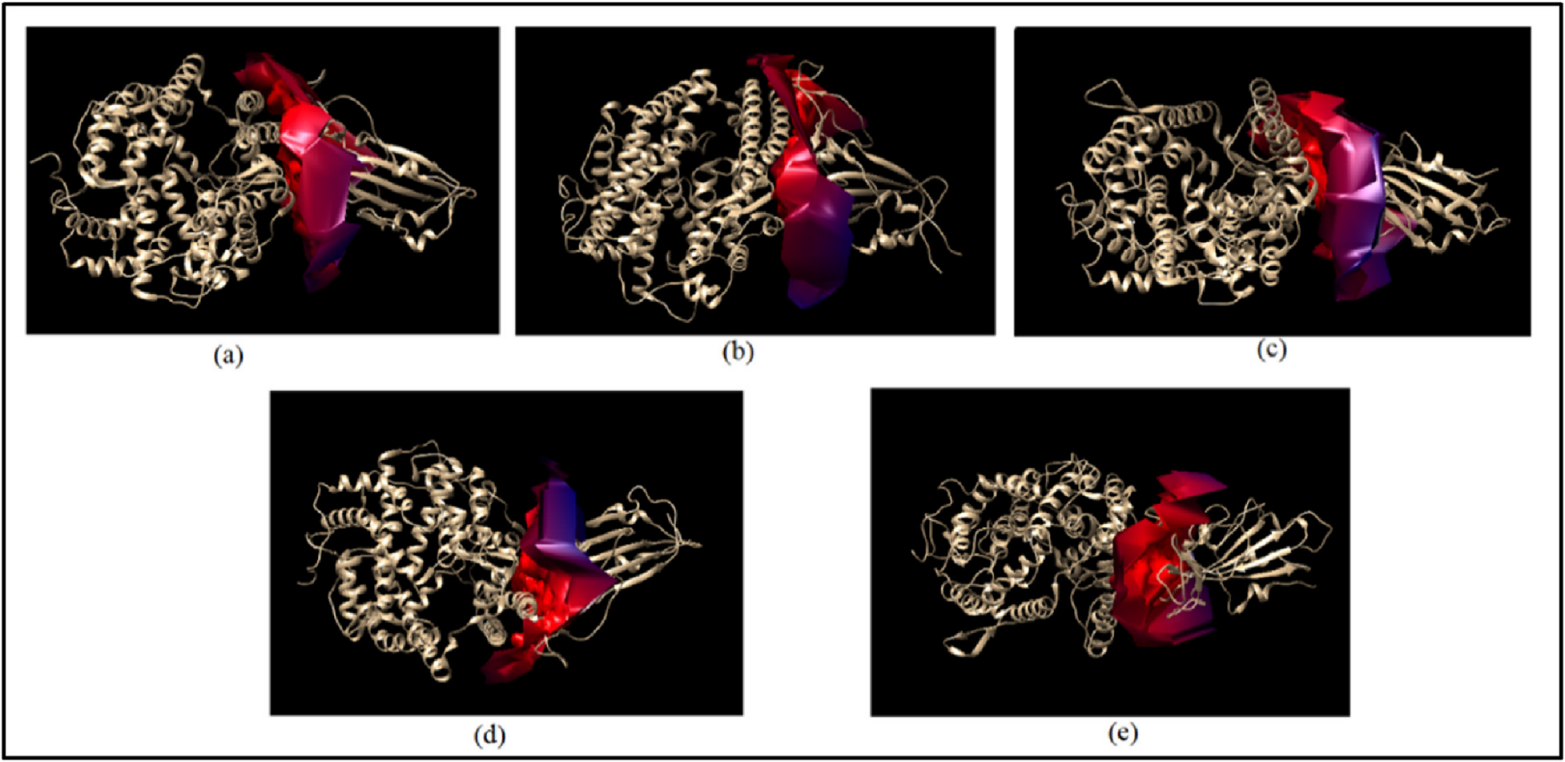
Interface surfaces between the investigated spike protein–ACE2 interfaces for the complexes of (a) 6M0J, (b) 7MJN, (c) 7LO4, (d) 7NXC and (e) 7EDJ.

Total free energies of the RBDs of isolated spike proteins, human ACE2 molecules and spike–ACE2 complexes are computed via density functional theory calculations and then binding free energies are obtained for comparison in this study. First of all, the atomic coordinate data of the original SARS-CoV-2–ACE2 complex, SARS-CoV-2 with N501Y mutant spike protein–ACE2 complex, SARS-CoV-2 with spike RBD having G485R mutation–ACE2 complex, SARS-CoV-2 P.1 variant spike glycoprotein–ACE2 complex and SARS-CoV-2 S-UK variant (B.1.1.7) with A570D and N501Y mutations–ACE2 complex are taken from the protein data bank (PDB) with IDs of 6M0J [23], 7MJN [24], 7LO4 [25], 7NXC [26] and 7EDJ [27], respectively. These mutations are selected according to their domination over other mutations at the time of this study. These protein data do not contain hydrogen atoms since Cryo-EM is used for obtaining these images. Therefore, hydrogens are added and additional molecules such as N-Acetylglucosamine (NAG) are cleaned as the first step. Then, the spike – ACE2 interfaces are determined by Chimera software [28,29] and then, all the atoms in the range of 15 Å of the interface surface are selected for DFT computations for the inclusion of longer-range quantum interaction effects. Therefore, a maximum of 30 Å range between the spike protein atoms and ACE2 molecule atoms are included in the calculations. The interface surfaces obtained in Chimera are shown in Figure 1.

**Figure 2:**
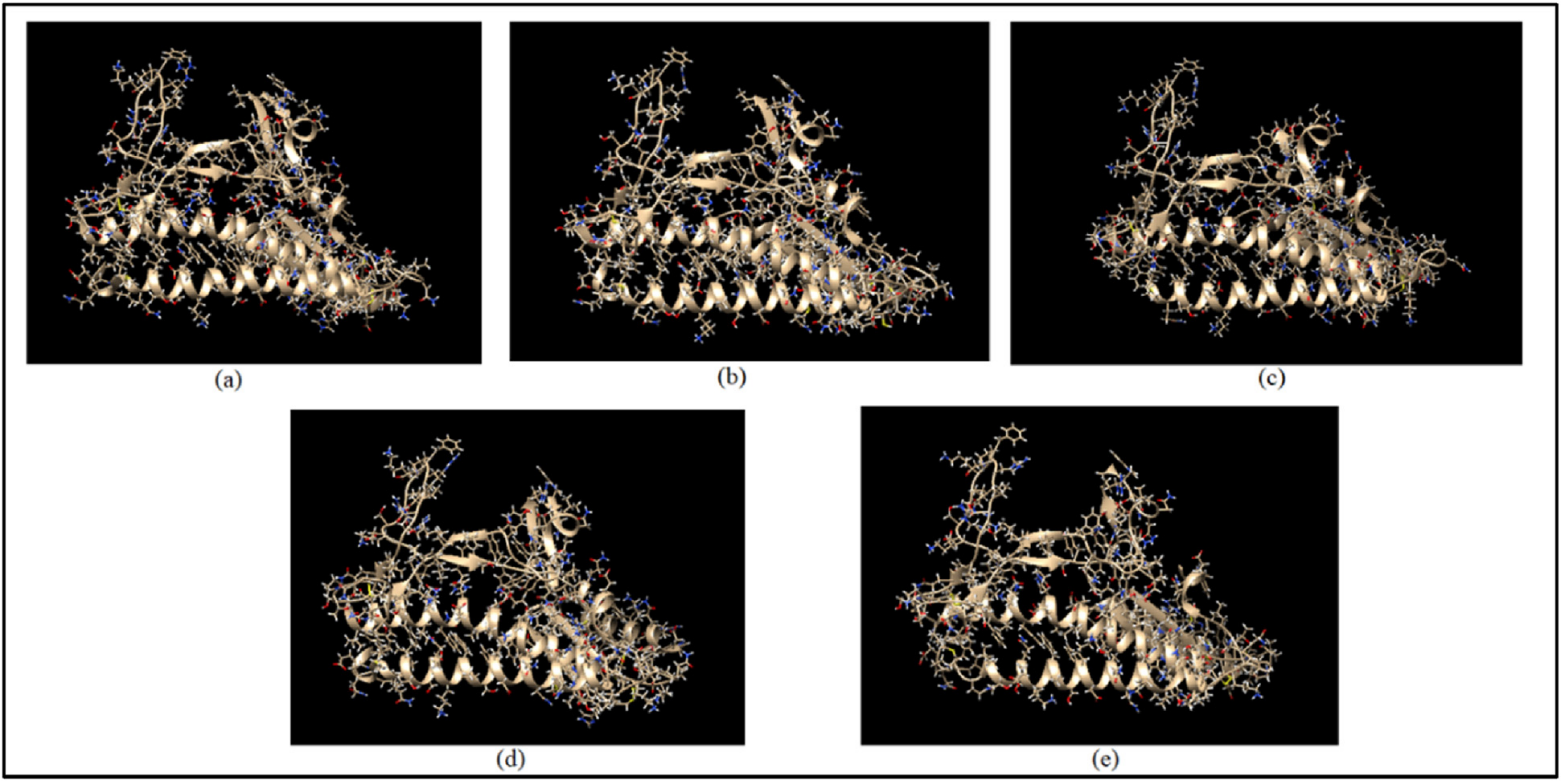
Obtained spike protein–ACE2 interfaces for the complexes of (a) 6M0J, (b) 7MJN, (c) 7LO4, (d) 7NXC and (e) 7EDJ via 15 Å neighbourhood of the interface surface.

The RBD of spike proteins and ACE2 molecules in the 15 Å neighbourhood that are selected with the intersurf operation [29] are also given in Figure 2. The number of atoms in each spike protein–ACE2 complex and isolated spike and ACE2 molecules are listed in Table 1.

**Table 1:** Number of atoms included in each spike–ACE2 complex and isolated molecules.

PDB ID	Number of atoms in spike–ACE2 complex	Number of atoms in the spike protein	Number of atoms in the ACE2 molecule
6M0J	3263	2061	1202
7MJN	3461	2276	1185
7LO4	3144	2027	1117
7NXC	3275	2099	1176
7EDJ	3223	2044	1179

As the first step, the spike protein–ACE2 complexes and isolated spike and ACE2 structures are firstly optimized before the actual DFT simulations. Optimization of these structures require large amount of RAM and processor power and these requirements are provided by the computing centre of the university of one of the authors (NNYU). The maximum step sizes has been set as 0.02 Å in the optimization phase with limited-memory Broyden-Fletcher-Goldfarb-Shanno (LBFGS) algorithm [30]. The average forces on the atoms of each structure after the optimization phase are given in Table 2. As it can be seen from Table 2, the forces are in an acceptable range to consider these structures as optimized.

**Table 2:** Average forces on the atoms of the investigated structures after the optimization step.

PDB ID	Average force on the atoms of the spike–ACE2 complex	Average force on the atoms of the spike protein structure	Average force on the atoms of the ACE2 molecule
6M0J	0.009 eV/Å	0.008 eV/Å	0.010 eV/Å
7MJN	0.007 eV/Å	0.007 eV/Å	0.012 eV/Å
7LO4	0.009 eV/Å	0.011 eV/Å	0.009 eV/Å
7NXC	0.012 eV/Å	0.014 eV/Å	0.012 eV/Å
7EDJ	0.006 eV/Å	0.008 eV/Å	0.008 eV/Å

DFT simulations for the computation of binding energies and partial charge densities at the spike protein–ACE2 interfaces are performed after the optimization phase. The gas phases of the structures are considered rather than the solvent phases since solvent phase interaction energy computations introduce higher uncertainties [16]. QuantumATK software is utilized for the DFT computations thanks to its effective algorithms [31,32]. The 15 Å neighbourhood of the spike protein–ACE2 interfaces are included in the spike–ACE2 complex computations without fragmentation to include the effects of longer-range electron interactions in the binding energy. Moreover, van der Waals dispersion corrections and counterpoise correction for basis set superposition errors (BSSE) are also included in the DFT simulations to increase accuracy [33,34]. It is worth noting that counterpoise correction requires about four times more computational cost as four different DFT computations are required. In addition to the total potential energy calculations, vibrational free energy computations are also required considering that the total free energy of a structure can be expressed as in Eq. (1) [35–39].

(1) $$G_{free}=H-TS$$

In Eq. (1), G_free_ is the Gibbs free energy, H is the enthalpy of the structure, T is the absolute temperature and S is the entropy. The enthalpy can be expressed as shown in Eq. (2).

(2) $$H=U_{cohesive}+\underset{U_{vib}}{\underbrace{U_{ZPE}+U_{THCE}}}+PV$$

In Eq. (2), U_cohesive_ is the cohesive potential energy, which is typically calculated as the total energy in a DFT simulation [30], UZPE, is the zero-point energy, U_THCE_ is the thermal correction energy for nonzero temperatures, P is the pressure and V is the volume [37, 38, 39]. The sum of U_ZPE_ and U_THCE_ can be abbreviated as the vibrational energy, U_vib_ [35]. It is worth noting that rotational and translational non-cohesive energy components are negligible compared to the vibrational energy. The cohesive energy, zero-point energy and thermal correction energy for nonzero temperatures can be expressed as in Eqs. (3), (4), and (5), respectively.

(3) $$U_{cohesive}\left[n\right]=T\left[n\right]+E_{XC}\left[n\right]+E_{H}\left[n\right]+E_{ext}\left[n\right]$$

(4) $$U_{ZPE}=-\frac{1}{N_{q}}\sum_{q,s}\frac{E\left(q,s\right)}{2}$$

(5) $$U_{THCE}=\frac{1}{N_{q}}\sum_{q,s}\frac{E\left(s,q\right)}{e^{\frac{E\left(s,q\right)}{k_{B}T}}-1}$$

In Eqs. (3), (4), and (5), n is the electron density, T[n] is the kinetic energy, E_XC_[n] is the exchange-correlation energy, E_H_[n] is the Hartree energy, E_ext_[n] is the interaction energy with an external field, N_q_ is the number of phonon modes, k_B_ is Boltzmann constant and E(q,s) denotes the phonon eigenenergies [37]. The cohesive energy is computed by DFT simulations, which is detailed in the previous paragraph. The zero-point energy and the thermal correction term in Eq. (2) includes vibrational contributions. The zero-point energy, thermal correction term and the entropy value are computed using the phonon density of states (DOS) hence making it possible to calculate the Gibbs free energies as expressed in Eq. (6). The non-cohesive part of the Gibbs free energy is abbreviated as U_non-cohesive_, which is the sum of the zero-point energy, thermal correction energy and the temperature times the entropy term as shown in Eq. (6).

(6) $$G_{free}=\underset{\left(computed\;from\;DFT\right)}{\underbrace{U_{cohesive}}}\underset{U_{non-cohesive}\;\left(computed\;from\;phonon\;DOS\right)}{\underbrace{\overset{U_{vib}}{\overset{︷}{-\frac{1}{N_{q}}\sum_{q,s}\frac{E\left(q,s\right)}{2}+\frac{1}{N_{q}}\sum_{q,s}\frac{E\left(q,s\right)}{e^{\frac{E\left(q,s\right)}{k_{B}T}-1}}}}-TS}}+PV$$

On the other hand, the binding free energy is defined as the difference of the bound and unbound Gibbs free energies as shown in Eq. (7) [40].

(7) $$\Delta G=G_{bound}-G_{unbound}$$

In Eq. (6), G_bound_ is the free energies of the bound states, G_unbound_ is the free energy of unbound states and ΔG is the binding free energy. For a spike protein–ACE2 complex, the binding free energy can be expressed as in Eq. (8) or equivalently as in Eq. (9).

(8) $$\Delta G_{binding}=G_{spike-ACE2\_complex}-G_{spike}-G_{ACE2}$$

(9) $$\Delta G_{binding}=\underset{\Delta U_{cohesive}}{\underbrace{U_{cohesive\_spike\_ACE2\_complex}-U_{cohesive\_spike}-U_{cohesive\_ACE2}}}+\underset{\Delta U_{vib}}{\underbrace{U_{vib\_spike\_ACE2\_complex}-U_{vib\_spike}-U_{vib\_ACE2}}}-\left(\underset{T\Delta S}{\underbrace{TS_{ACE2-spike\_complex}-TS_{spike}-TS_{ACE2}}}\right)+\Delta PV$$

In Eq. (8), ΔG_binding_ is the binding free energy of the spike protein–ACE2 complex while G_spike-ACE2_complex_, G_spike_ and G_ACE2_ denote the Gibbs free energies of the spike protein–ACE2 complex, isolated spike protein and ACE2 structures, respectively. Hence, using Eq. (9), the binding free energies of the investigated spike–ACE2 complexes are calculated using the cohesive potential energies obtained from DFT simulations together with zero-point energies, thermal correction energies and entropy values computed from the phonon density of states considering that ΔPV is zero for constant number of particles [36].

## 3 Results and discussion

In order to apply the methodology explained in the previous section, DFT simulations are performed in QuantumATK software [31]. In all of the DFT simulations, double-zeta polarized (DZP) basis set [41,42] with generalized gradient approximation (GGA) exchange-correlation functional [43] are used for accuracy and optimal simulation cost. The mesh cut-off energy is taken as 100Ry (1360.56 eV) and the energy iteration tolerance is selected as 10^−4^ eV to achieve precision within the memory limits of the computational cluster. The peak memory requirement with 24 processors was more than 400 GB in the DFT computation of the spike protein–ACE2 complex with the selection of these simulation parameters, which takes four different DFT iterations with the inclusion of counterpoise correction as explained before. It is worth noting that the same simulation parameters are used for all of the considered structures to perform the proper comparison of the binding free energies. The cohesive energies obtained from DFT simulations, non-cohesive energies computed from phonon DOS and the resulting Gibbs free energies of the considered spike protein–ACE2 complexes, isolated spike protein and isolated ACE2 structures are computed for two different temperatures of 36.5 °C and 39 °C using Eq. (6) and presented in Table 3. The reason for the calculation of Gibbs free energies for 36.5 °C and 39 °C is to check if there is any difference in the binding free energies at the normal body temperature of 36.5 °C and an elevated temperature of 39 °C, which may occur after the incubation period. This approach obviously doubled the computation cost of the calculation of the non-cohesive energies.

**Table 3:** Cohesive energies, non-cohesive energies and the resulting Gibbs free energies of the considered structures at 36.5 °C and 39 °C.

Structure ID	Cohesive energy (eV)	Non-cohesive energy @ 36.5 °C (eV)	Non-cohesive energy @ 39 °C (eV)	Gibbs free energy @ 36.5 °C (eV)	Gibbs free energy @ 39 °C (eV)
6M0J spike–ACE2 complex	−417264.51597	898.03484	897.31963	−416366.48113	−416367.19634
6M0J spike protein only	−152304.31800	329.55357	329.29595	−151974.76443	−151975.02205
6M0J ACE2 protein only	−264954.91424	566.03293	565.57785	−264388.88131	−264389.33639
7MJN spike–ACE2 complex	−441438.82759	954.81032	954.058518	−440484.01727	−440484.76907
7MJN spike protein only	−150158.34828	327.94766	327.70162	−149830.40062	−149830.64666
7MJN ACE2 protein only	−291271.83015	626.76516	626.26494	−290645.06499	−290645.56521
7LO4 spike–ACE2 complex	−403227.85806	867.05023	866.36074	−402360.80783	−402361.49732
7LO4 spike protein only	−141878.93599	308.68014	308.44195	−141570.25585	−141570.49404
7LO4 ACE2 protein only	−261340.87563	556.38815	555.93866	−260784.48748	−260784.93697
7NXC spike–ACE2 complex	−414031.94856	904.76476	904.05264	−413127.18380	−413127.89592
7NXC spike protein only	−148189.43655	324.99608	324.74879	−147864.44047	−147864.68776
7NXC ACE2 protein only	−265833.40065	579.64581	579.18865	−265253.75484	−265254.21200
7EDJ spike–ACE2 complex	−412803.67110	886.78218	886.07945	−411916.88892	−411917.59165
7EDJ spike protein only	−149774.58397	325.09593	324.84833	−149449.48804	−149449.73564
7EDJ ACE2 protein only	−263012.72073	561.50945	561.06209	−262451.21128	−262451.65864

The binding free energies of the spike protein–ACE2 complexes are then calculated using Eq. (8) and given in Table 4. The following points are observed from Table 4: i) all the binding free energies are negative meaning that spike–ACE2 attachment reactions are spontaneous, ii) the binding free energies of the same spike–ACE2 complex are slightly lower at 39 °C compared to the binding free energies at 36.5 °C implying that the spike–ACE2 attachment reaction would become slightly more favourable at 39 °C, iii) the binding free energy of the mutated SARS-CoV-2–ACE2 structures investigated in this study have lower values compared to that of the original strain (6M0J), which would aid explaining why these mutated SARS-CoV-2 variants have the tendency to take over the original strain, and iv) the binding free energy of the SARS-CoV-2 S-UK variant (B.1.1.7) with A570D and N501Y mutations–ACE2 complex (7EDJ) has the lowest value meaning that SARS-CoV-2 S-UK variant is the most favourable strain, as also observed by the significant spread of this variant [13,44]. It is also worth comparing the obtained binding free energies with the values existing in the literature. There are two studies employing DFT with van der Waals dispersion correction, which consider the binding free energies of the spike–ACE2 complexes of coronaviruses to the knowledge of the authors. One of the studies of Rodriguez and Gupta reported the binding free energy of SARS-CoV-1–ACE2 in the range of –340.46 kcal/mol to –404.26 kcal/mol for different basis sets [16]. Another study by Rodriguez investigated the binding free energies of the fragments of the original strain of SARS-CoV-2 spike–ACE2 complex as reported in Tables S1 to S4 of the supplementary material of [17] where the total binding free energies of the fragments result between –42.02 kcal/mol to –98.60 kcal/mol depending on the basis set employed in DFT simulations. Therefore, it can be stated that the binding free energy values obtained in our study are compliant with the order of the binding free energies existing in the literature that are computed using DFT with dispersion correction. However, it is again worth noting that our study includes basis set superposition error corrections and the inclusion of non-cohesive energies.

**Table 4:** Binding free energies of the investigated spike protein–ACE2 complexes.

Structure ID	Binding free energy @ 36.5 °C	Binding free energy @ 39 °C
6M0J	−2.83538 eV (−65.38386 kcal/mol)	−2.83790 eV (−65.44197 kcal/mol)
7MJN	−8.55166 eV (−197.20127 kcal/mol)	−8.55719 eV (−197.32880 kcal/mol)
7LO4	−6.06450 eV (−139.84737 kcal/mol)	−6.06631 eV (−139.88910 kcal/mol)
7NXC	−8.98849 eV (−207.27457 kcal/mol)	−8.99615 eV (−207.45121 kcal/mol)
7EDJ	−16.18959 eV (−373.33194 kcal/mol)	−16.19737 eV (−373.51135 kcal/mol)

**Figure 3:**
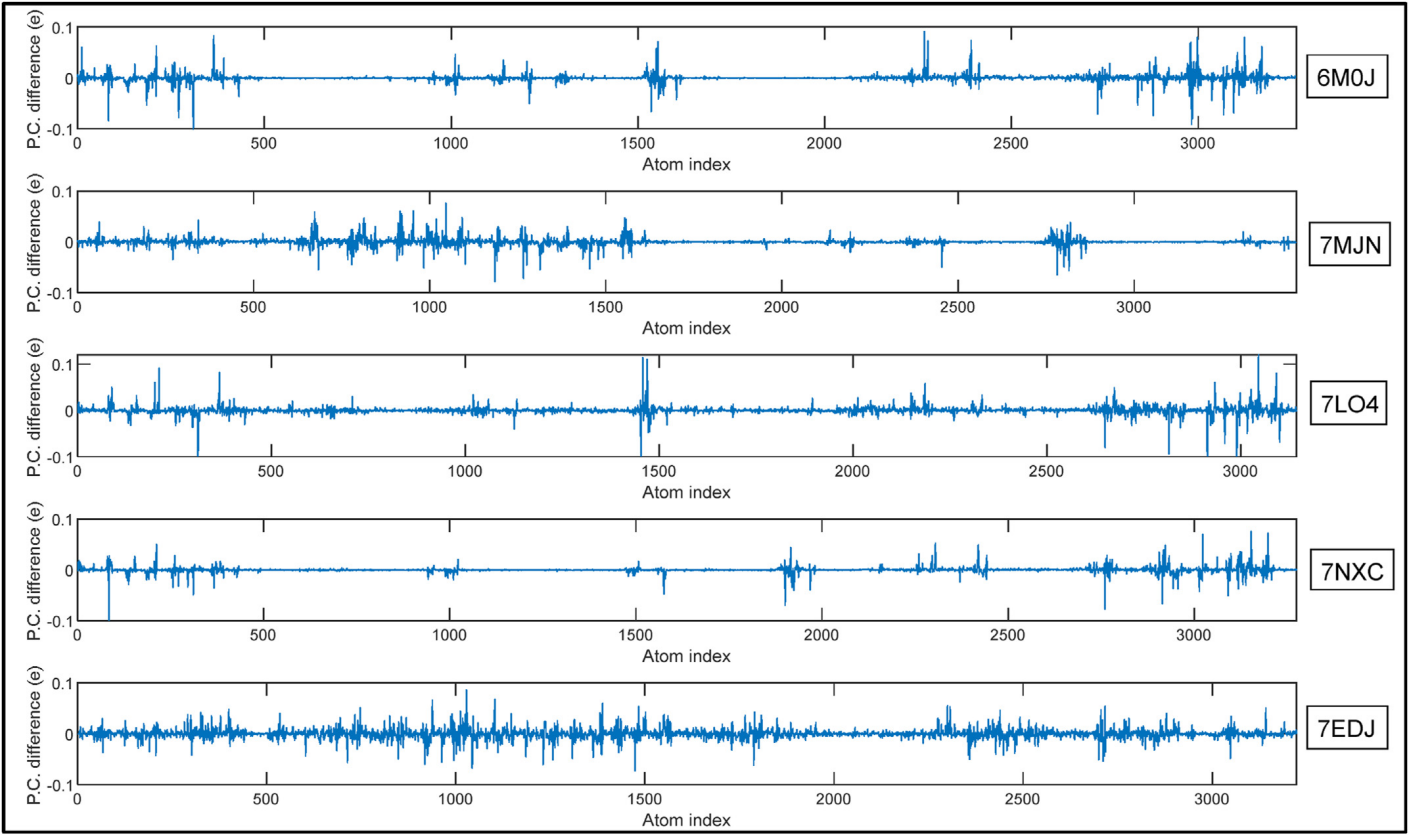
Partial charge changes of the atoms in the investigated spike–ACE2 complexes before and after the attachment processes.

**Figure 4:**
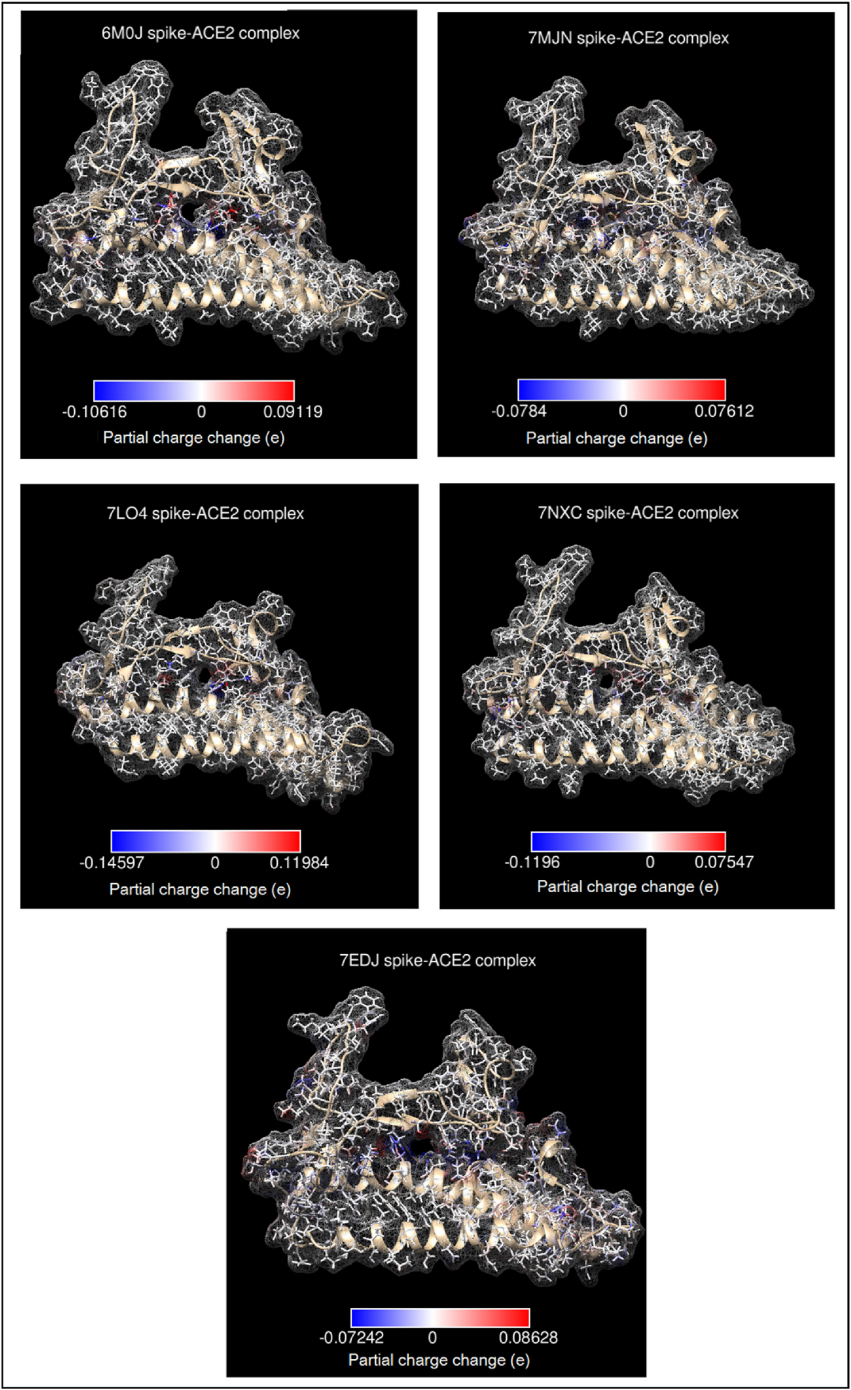
3D depiction of the partial charge changes of the investigated spike–ACE2 complexes before and after the attachment processes.

In order to interpret the differences of the binding properties of the investigated complexes further, we have extracted the partial electronic charge changes of spike and ACE2 structures and the spike–ACE2 complexes before and after the attachment reactions. The total partial charge differences are found to be 5.982^−^e, 5.991^−^e, 7.069^−^e, 4.188^−^e and 10.421^−^e for the complexes with IDs of 6M0J, 7MJN, 7LO4, 7NXC and 7EDJ, respectively. The spread of the partial charge differences among atoms are obtained and plotted as shown in Figure 3. The higher number of atoms having their partial charges changed after the attachment process of the SARS-CoV-2 S-UK variant (B.1.1.7) (7EDJ) to the ACE2 is clearly observed from Figure 3. Computed partial charge differences are parsed to the appropriate format and then imported to the Chimera software as attribute assignment files and visualized in 3D as shown in Figure 4. The higher spread of the partial charge difference of the SARS-CoV-2 S-UK variant (B.1.1.7) with A570D and N501Y mutations (7EDJ)–ACE2 complex can also be observed in 3D as in Figure 4. It is worth noting that the partial charge changes give clues about the atomic contributions to the attachment process but it is not the whole story as van der Waals dispersion forces [16,17] have significant effect on the binding free energies as well as the vibrational energies as included in our study.

## 4 Conclusions

Original strain and various mutations of the SARS-CoV-2 spike protein–human ACE2 complexes are considered in this study and their binding free energies are obtained and compared using density functional theory calculations. The investigated variants are SARS-CoV-2 with N501Y mutant spike protein, spike RBD having G485R mutation, SARS-CoV-2 P.1 variant spike protein mutation and the S-UK variant (B.1.1.7) with A570D and N501Y mutations. In order to include the longer-range electron-electron and electron-ion interactions in the DFT calculations, all the atoms in the 15 Å range of the spike–ACE2 interfaces are included in computations without fragmentation. Basis set superposition error correction schemes as well as van der Waals dispersion calculations are utilized in DFT simulations. Moreover, the vibrational free energies via the computation of phonon density of states and dynamical matrices are also incorporated for the calculation of Gibbs free energies. The results show that the absolute value of the binding free energy of the S-UK variant (B.1.1.7) SARS-CoV-2 with A570D and N501Y mutations has the highest value, being more than five times higher than that of the original strain, which may aid explaining the spread of this variant. The absolute values of the binding free energies of the other three investigated SARS-CoV-2 spike protein variants to ACE2 are also two to three times greater than that of the original strain. In addition, the vibrational free energies of the investigated complexes are calculated for two temperatures, 36.5 °C and 39 °C as the euthermia and hyperthermia points. The binding free energies at 39 °C are found to be slightly lower than the values at 36.5 °C for the same structure, which is interesting for comparing the attachment of spike proteins to ACE2 at normal body temperatures and elevated body temperatures. In order to investigate the binding free energy differences of the considered structures further, the partial charge differences of the atoms before and after the spike–ACE2 attachment process in each structure are calculated. It is exposed that the total partial charge difference of the S-UK variant (B.1.1.7) SARS-CoV-2 with A570D and N501Y mutations is higher and more spread among atoms meaning that more number of atoms change their charges during the spike – ACE2 attachment for this variant. The results of this study is expected to be useful for understanding the spike–ACE2 attachment properties and the methodology utilized for calculating binding free energies using DFT and phonon DOS is considered to be promising to estimate the spreading potential of upcoming SARS-CoV-2 variants.

## Declarations

### Author contribution statement

Serhan Yamacli: Conceived and designed the experiments; Performed the experiments; Contributed reagents, materials, analysis tools or data; Wrote the paper.

Mutlu Avci: Conceived and designed the experiments; Performed the experiments; Analyzed and interpreted the data; Wrote the paper.

### Funding statement

This research did not receive any specific grant from funding agencies in the public, commercial, or not-for-profit sectors.

### Data availability statement

Data will be made available on request.

### Declaration of competing interest

The authors declare no conflict of interest.

### Additional information

No additional information is available for this paper.
